# Supplementary material for: The Influence of OAT1 Density and Functionality on Indoxyl Sulfate Transport in the Human Proximal Tubule: An Integrated Computational and In Vitro Study
Source: Toxins (Basel). 2021 Sep 22;13(10):674. doi: 10.3390/toxins13100674 (PMC8538816; doi:10.3390/toxins13100674)
Supplement: Supplementary file 1 [file toxins-13-00674-s001.zip › toxins-1321471-supplementary.pdf]

The Influence of OAT1 Density and Functionality on Indoxyl Sulfate Transport in the Human Proximal Tubule: An Integrated Computational and In Vitro Study

Jasia King, Silvia M. Mihaila, Sabbir Ahmed, Roman Truckenmüller, Stefan Giselbrecht, Rosalinde Masereeuw and Aurélie Carlier

Table S1: Conservation of mass calculation

Time Point	Toxin in Blood	OAT bound Toxin	OAT1 Unbound	Toxin in Dialysate	Total Toxin	Total OAT1
Start	1.51×10^{14}	0.00×10^{0}	3.68×10^{14}	0×10^{0}	$\mathbf{1.51 \times 10^{14}}$	$\mathbf{3.68 \times 10^{14}}$
Steady State	1.22×10^{14}	2.87×10^{13}	3.39×10^{14}	2.16×10^{11}	$\mathbf{1.51 \times 10^{14}}$	$\mathbf{3.68 \times 10^{14}}$

Table S2. All settings for COPASI parameter fitting models.

COPASI Parameter Fitting Settings for Guessing Fitted Values for the Parameter Fitting Model				
Parameter		Initial Guess	Lower Limit	Upper Limit
Case 1	kf_{Uptake} [$s^{-1}.\mu M^{-1}$] IS binding to transporter	1×10^{-4}	5×10^{-7}	1×10^{-3}
	$kf_{dissociation}$ [s^{-1}] IS dissociation from transporter	1×10^{-3}	5×10^{-5}	1×10^{-3}
	[OAT1] [$molecules.\mu m^{-2}$] Density of transporter	5×10^{6}	7×10^{5}	2×10^{7}
Case 3	$kf_{dissociation}$ [s^{-1}] IS dissociation from transporter	4.18×10^{-4}	4×10^{-4}	1×10^{-3}
	K_M	1×10^{2}	1×10^{0}	1×10^{3}
	V_{Max} [s^{-1}]	1×10^{-1}	1×10^{-5}	1×10^{1}
Case 3	$kf_{dissociation}$ [s^{-1}] IS dissociation from transporter	1.69×10^{-4}	1.69×10^{-5}	1.69×10^{-3}
	K_M	1×10^{0}	1×10^{-3}	1×10^{3}
	V_{Max} [s^{-1}]	1×10^{0}	1×10^{-6}	1×10^{2}
